# Cardiovascular benefit in the limelight: shifting type 2 diabetes treatment paradigm towards early combination therapy in patients with overt cardiovascular disease

**DOI:** 10.1186/s12933-018-0760-6

**Published:** 2018-08-22

**Authors:** Naim Shehadeh, Itamar Raz, Afif Nakhleh

**Affiliations:** 1 Institute of Endocrinology, Diabetes and Metabolism, Rambam Health Care Campus, HaAliya HaShniya Street 8, 3109601 Haifa, Israel; 2 Diabetes Unit, Hadassah Hebrew University Hospital, Jerusalem, Israel

**Keywords:** Diabetes mellitus type 2, Mild hyperglycemia, Early combination therapy, Cardiovascular disease, Cardiovascular outcomes

## Main text

The leading cause of morbidity and mortality in patients with diabetes mellitus is cardiovascular disease (CVD).

In the EMPA-REG OUTCOME [1] and the CANVAS Program [2], the sodium–glucose co-transporter 2 (SGLT2) inhibitors empagliflozin and canagliflozin reduced the major cardiovascular (CV) adverse events among patients with diabetes mellitus type 2 (T2DM) and established CVD or at high risk for CV events. Reduction in heart failure hospitalizations and improved renal outcomes were also demonstrated, although these findings were not considered as statistically significant for the CANVAS Program. Empagliflozin in the EMPA-REG OUTCOME, but not canagliflozin in the CANVAS Program, remarkably reduced all-cause and CV mortality. The United States Food and Drug Administration (FDA) recently approved empagliflozin for reducing cardiovascular mortality in adults with T2DM and established CVD. In the LEADER and SUSTAIN 6 trials, the glucagon-like peptide-1 receptor agonists (GLP-1 RAs) liraglutide and once-weekly semaglutide, significantly decreased the risk for major CV adverse events among T2DM patients with established CVD or more than one CV risk factor [3, 4]. In SUSTAIN 6, a significant decrease in the rate of nonfatal stroke was observed in the semaglutide group, with no significant reduction in the rate of cardiovascular death. In LEADER, liraglutide significantly reduced CV and all-cause mortality. A lower rate of nephropathy events was observed in the liraglutide group compared to placebo [5]. Liraglutide recently received FDA approval for reducing the risk of major adverse CV events, including nonfatal myocardial infarction, nonfatal stroke, and CV mortality in adults with T2DM and established CVD.

Current diabetes clinical practice guidelines [6–8] recommend lifestyle modification and metformin, if not contraindicated, as the preferred initial pharmacologic agent for the treatment of patients with recent onset T2DM and mild hyperglycemia. For patients who present with higher HbA1c levels, ADA guidelines advocate a stepwise approach that intensifies treatment incrementally to dual and triple therapy at 3-month intervals until the patients are at their individualized goal, taking into consideration initial dual therapy if HbA1c ≥ 9% at diagnosis [6]. AACE/ACE guidelines encourage initial dual therapy for patients who present with HbA1c > 7.5% at diagnosis, with a suggested hierarchy of use in which GLP-1 RA ranks second and SGLT2-2i ranks third after metformin [7]. Similarly, Diabetes Canada guidelines state that initiating metformin in combination with a second glucose-lowering agent should be considered if HbA1c values are ≥ 1.5% above target at diagnosis [8]. Recent guidelines address the FDA approval of empagliflozin and liraglutide for CVD benefit. The ADA and Diabetes Canada guidelines recommend that in patients with T2DM and established CVD, antihyperglycemic therapy should begin with lifestyle management and metformin and subsequently incorporate an agent proven to reduce major adverse CV events and CV mortality, currently empagliflozin and liraglutide (canagliflozin may be considered) after considering drug-specific and patient factors. Notably, the ADA and Diabetes Canada guidelines discern drug-specific CV effects into atherosclerotic cardiovascular disease (ASCVD) and heart failure (HF) effects that should be considered when selecting treatment, along with renal dosing considerations. All guidelines endorse individualized glycemic targets based on numerous factors, such as life expectancy, comorbid conditions, duration of diabetes, and risk of hypoglycemia. AACE/ACE supports an HbA1c level of ≤ 6.5% for most patients, but higher targets may be appropriate if lower HbA1c cannot be achieved without adverse outcomes. ADA guidelines denote HbA1c of 7% as a reasonable goal for many nonpregnant T2DM patients and suggest more stringent targets (such as HbA1c < 6.5%) for selected patients at low risk for hypoglycemia or other adverse effects and patients with long-life expectancy and no significant CVD. Less stringent HbA1c goals (such as HbA1c < 8%) may be appropriate for patients with a history of severe hypoglycemia or advanced microvascular or macrovascular complications.

The current guidelines [6–8] do not clearly address the implementation of early combination therapy of metformin and an agent with evidence of CV risk reduction in patients with recent onset T2DM and established CVD who present with mild hyperglycemia (defined as HbA1c < 7.5%).

In light of the demonstrated CV benefit with empagliflozin and liraglutide in a manner that seems unlikely to be attributed to glycemic control alone and their recent FDA approval for this indication, we would like to propose an important modification to the current T2DM treatment paradigm that primarily considers CVD risk profile at earlier stages of T2DM. We suggest preferentially combining metformin with a glucose-lowering agent proven to reduce major adverse CV events and CV mortality (currently empagliflozin or liraglutide) in T2DM patients that present with mild hyperglycemia (HbA1c < 7.5%) and match the eligibility criteria of EMPA-REG OUTCOME and LEADER trials for established ASCVD, including significant coronary artery disease, myocardial infarction, ischemic stroke, transient ischemic attack, and peripheral artery disease and HF.

Several considerations should be sought in preferring one agent over another, regarding the benefits and harms of interventions in congruence with the prevalent evidence in literature.

Liraglutide (at a daily dose of 1.8 mg) should be considered in patients with overt CVD, obesity and chronic kidney disease stage 3 or 4. Empagliflozin (at a daily dose of 10 mg or 25 mg) should be considered in patients who have overt CVD and HF or are at high risk of developing HF. We suggest that metformin may be initiated at a submaximal dose (given the mild hyperglycemia) and optimized individually based on glycemic control and renal function.

Our suggestion for early combination of metformin and a glucose-lowering agent with a proven cardiovascular benefit among T2DM patients with overt CVD is inspired by previous findings. In a nationwide primary care database survey in Denmark, the prevalence of CVD in patients with T2DM was 21.4%, the mean HbA1c of patients with type 2 diabetes and CVD was 6.9%, and 23.5% of them had HbA1c > 7.5% [9]. If extrapolated to other patient populations, this implies that a considerable portion of our T2DM patients with overt CVD have HbA1c < 7.5%, and they would benefit from early initiation of antihyperglycemic agents with a proven CV efficacy.

There is some evidence in literature that this benefit might extend to T2DM patients with milder degrees of hyperglycemia. A recent post hoc analysis has shown that in the EMPA REG OUTCOME trial, the reduction in CV death with empagliflozin appeared to occur irrespective of baseline HbA1c or the change in HbA1c from baseline to the last value in the trial [10]. In LEADER trial, there was no significant difference in terms of primary composite CV outcome between baseline HbA1c of ≤ 8.3% and > 8.3% groups [3].

Another evidence comes from several studies that have emphasized the importance of early treatment in diabetes, not only to prevent microvascular complications, but also to prevent CV complications years after medication inception, as has been observed in the UKPDS trial after 20 years of observational follow-up, referred to as a “legacy effect” [11].

Furthermore, SGLT2 inhibitors and GLP-1 RAs may provide additive effects in reducing blood pressure and body weight and preserving renal function, an observation of particular relevance to T2DM patients at high CV risk [12, 13]. Both EMPA-REG and CANVAS Program trials demonstrated salutary effects of SGLT2 inhibitors on the kidney, including reduced progression of albuminuria and lower rates of clinically relevant renal events [2, 14]. Liraglutide in LEADER, significantly reduced nephropathy events, although this was predominantly driven by reduction in new-onset persistent macroalbuminuria [5].

Finally, while there is robust evidence for the efficacy of metformin on glycemic control and wide clinical experience support overall and CV safety, the CV efficacy of metformin remains uncertain. It should be noted that major conclusions regarding metformin benefit on selected CV outcomes were drawn from the UKPDS trial [15] and these observations have not been replicated in prospective trials, limiting the generalizability of results across T2DM patients with overt CVD.

We believe that our suggested early combination approach would lead to a prominent decrease in CV events among T2DM patients with overt CVD. However, limitations of current evidence should be kept in mind; recent CV outcome trials [1–4] have enrolled relatively small percentages of patients with mild hyperglycemia (HbA1c < 7.5%), HF, or moderate renal impairment (CKD stage 3). Ongoing and further well-designed studies should assess and clarify the renal and CV efficacy and safety profile of liraglutide and empagliflozin across these groups of patients.

## Abbreviations

CVD: cardiovascular disease
SGLT-2: sodium–glucose co-transporter 2
CV: cardiovascular
T2DM: diabetes mellitus type 2
FDA: United States Food and Drug Administration
GLP-1 RA: glucagon-like peptide-1 receptor agonist
ASCVD: atherosclerotic cardiovascular disease
HF: heart failure
